# Prediction of microRNA-disease associations based on distance correlation set

**DOI:** 10.1186/s12859-018-2146-x

**Published:** 2018-04-17

**Authors:** Haochen Zhao, Linai Kuang, Lei Wang, Pengyao Ping, Zhanwei Xuan, Tingrui Pei, Zhelun Wu

**Affiliations:** 1 College of Computer Engineering & Applied Mathematics, Changsha University, Changsha, 410001 Hunan People’s Republic of China; 2 Key Laboratory of Intelligent Computing & Information Processing (Xiangtan University), Ministry of Education, China, Xiangtan, 411105 Hunan People’s Republic of China; 3 Department of Computer Science, Lakehead University, Thunder Bay, ON P7B5E1 Canada; 4 Department of Computer Science, Princeton University, Princeton, New Jersey USA; 5 College of Information Engineering, Xiangtan University, Xiangtan, Hunan People’s Republic of China

**Keywords:** MiRNA-disease association predictions, Distance correlation set, Disease-lncRNA-miRNA network, Similarity measure

## Abstract

**Background:**

Recently, numerous laboratory studies have indicated that many microRNAs (miRNAs) are involved in and associated with human diseases and can serve as potential biomarkers and drug targets. Therefore, developing effective computational models for the prediction of novel associations between diseases and miRNAs could be beneficial for achieving an understanding of disease mechanisms at the miRNA level and the interactions between diseases and miRNAs at the disease level. Thus far, only a few miRNA-disease association pairs are known, and models analyzing miRNA-disease associations based on lncRNA are limited.

**Results:**

In this study, a new computational method based on a distance correlation set is developed to predict miRNA-disease associations (DCSMDA) by integrating known lncRNA-disease associations, known miRNA-lncRNA associations, disease semantic similarity, and various lncRNA and disease similarity measures. The novelty of DCSMDA is due to the construction of a miRNA-lncRNA-disease network, which reveals that DCSMDA can be applied to predict potential lncRNA-disease associations without requiring any known miRNA-disease associations. Although the implementation of DCSMDA does not require known disease-miRNA associations, the area under curve is 0.8155 in the leave-one-out cross validation. Furthermore, DCSMDA was implemented in case studies of prostatic neoplasms, lung neoplasms and leukaemia, and of the top 10 predicted associations, 10, 9 and 9 associations, respectively, were separately verified in other independent studies and biological experimental studies. In addition, 10 of the 10 (100%) associations predicted by DCSMDA were supported by recent bioinformatical studies.

**Conclusions:**

According to the simulation results, DCSMDA can be a great addition to the biomedical research field.

**Electronic supplementary material:**

The online version of this article (10.1186/s12859-018-2146-x) contains supplementary material, which is available to authorized users.

## Background

For a long time, RNA was considered a DNA-to-protein gene sequence transporter [1]. The sequencing of the human genome indicates that only approximately 2% of the sequences in human RNA are used to encode proteins [2]. Furthermore, numerous studies performing biological experiments have indicated that noncoding RNA (ncRNA) plays an important role in numerous critical biological processes, such as chromosome dosage compensation, epigenetic regulation and cell growth [3–5]. MicroRNAs (miRNAs) are endogenous single-stranded ncRNA molecules approximately 22 nt in length that regulate the expression of target genes by base pairing with the 3′-untranslated regions (UTRs) of the target genes [6, 7]. Recently, several studies have reported that more than one-third of genes are regulated by miRNAs [8], and more than 1000 miRNAs have been identified using various experimental methods and computational models [9, 10]. In addition, accumulating evidence indicates that many microRNAs (miRNAs) are involved in and associated with human diseases, such as myocardial disease, Alzheimer’s disease, cardiovascular disease and heart disease [11–14]. Therefore, identifying disease-miRNA associations could not only improve our knowledge of the underlying disease mechanism at the miRNA level but also facilitate disease biomarker detection and drug discovery for disease diagnosis, treatment, prognosis and prevention. However, compared with the rapidly increasing number of newly discovered miRNAs, only a few miRNA-disease associations are known [15, 16]. Developing efficient, successful computational approaches that predict potential miRNA-disease associations is challenging and urgently needed.

Recently, several heterogeneous biological datasets, such as HMDD and miR2Disease, have been constructed [17–19], and several computational methods are used to predict potential miRNA-disease associations based these datasets [20–22]. For example, Jiang et al. developed a scoring system to assess the likelihood that a microRNA is involved in a specific disease phenotype based on the assumption that functionally related microRNAs tend to be associated with phenotypically similar diseases [23]. K. Han et al. developed a prediction method called DismiPred that combines functional similarity and common association information to predict potential miRNA-disease associations based on the central hypothesis offered in several previous studies that miRNAs with similar functions are often involved in similar diseases [24]. Furthermore, Xuan et al. proposed a method called HDMP to predict potential disease-miRNA associations based on weighted k most similar neighbours [25] and developed a method for predicting potential disease-associated microRNAs based on random walk (MIDP) [26]. Chen et al. proposed a prediction method called RWRMDA by implementing random walk on the miRNA functional similarity network and further proposed a model called RLSMDA based on semi-supervised learning by integrating a disease-disease semantic similarity network, miRNA-miRNA functional similarity network, and known human miRNA-disease associations for the prediction of potential disease-miRNA associations [27]. In 2016, based on the assumption that functionally similar miRNAs tend to be involved in similar diseases, Chen et al. developed a prediction model called WBSMDA by integrating known miRNA-disease associations, miRNA functional similarity networks, disease semantic similarity networks, and Gaussian interaction profile kernel similarity networks to uncover potential disease-miRNA associations [28].

In the abovementioned computational models, known miRNA-disease associations are required. However, few lncRNA-disease associations have been recorded in several biological datasets, such as MNDR and LncRNADisease [29, 30], and several studies have shown that lncRNA-miRNA associations are involved in and associated with human diseases [31–33]. Thus, in this article, a new model based on the Distance Correlation Set for MiRNA-Disease Association inference (DCSMDA) was developed to predict potential miRNA-disease associations by integrating known lncRNA-disease and lncRNA-miRNA associations, the semantic similarity and functional similarity of the disease pairs, the functional similarity of the miRNA pairs, and the Gaussian interaction profile kernel similarity for the lncRNA, miRNA and disease. Compared with existing state-of-the-art models, the advantage of DCSMDA is its integration of the similarity of the disease pairs, lncRNA pairs, miRNA pairs, and introduction of the distance correlation set; thus, DCSMDA does not require known miRNA-disease associations. Moreover, leave-one-out cross-validation (LOOCV) was implemented to evaluate the performance of DCSMDA based on known miRNA-disease associations downloaded from the HMDD database, and DCSMDA achieved a reliable area under the ROC curve (AUC) of 0.8155. Moreover, case studies of lung neoplasms, prostatic neoplasms and leukaemia were implemented to further evaluate the prediction performance of DCSMDA, and 9, 10 and 9 of the top 10 predicted associations in these three important human complex diseases have been confirmed by recent biological experiments. In addition, a case study identifying the top 10 lncRNA-disease associations showed that 10 of the 10 (100%) associations predicted by DCSMDA were supported by recent bioinformatical studies and the latest HMDD dataset, effectively demonstrating that DCSMDA had a good prediction performance in inferring potential disease-miRNA associations.

## Results

To evaluate the prediction performance of DCSMDA, first, our method was compared with other state-of-the-art methods in the framework of the LOOCV, and then, we analyzed the stability of DCSMDA using three lncRNA-disease datasets. Second, we analyzed the effect of the pre-determined threshold parameter *b*. Finally, several additional experiments were performed to validate the feasibility of our method.

### Performance comparison with other methods

Since our method is unsupervised (i.e., known miRNA-disease associations are not used in the training) and the few proposed prediction models for the large-scale forecasting of the associations between miRNAs and diseases are simultaneously based on known miRNA-lncRNA associations and known lncRNA-disease associations, to validate the prediction performance of our novel model, we compared the prediction performance of DCSMDA with that of three state-of-the-art computational prediction models, including WBSMDA [28], RLSMDA [27] and HGLDA [31]; WBSMDA and RLSMDA are semi-supervised methods that do not require any negative samples, and HGLDA is an unsupervised method developed to predict potential lncRNA-disease associations by integrating known miRNA-disease associations and lncRNA-miRNA interactions.

To compare the performance of DCSMDA with that of WBSMDA and RLSMDA, we adopted the *DS*_5_ dataset and the framework of the LOOCV. While the LOOCV was implemented for these three methods, each known miRNA-disease association was left out in turn as the test sample, and we further evaluated how well this test association ranked relative to the candidate sample. Here, the candidate samples comprised all potential miRNA-disease associations without any known association evidence. Then, the testing samples with a prediction rank higher than the given threshold were considered successfully predicted. If the testing samples with a prediction rank higher than the given threshold were considered successfully predicted, then DCSMDA, RLSMDA and WBSMDA were checked in the LOOCV.

To compare the performance of DCSMDA with that of HGLDA, we adopted the *DS*_3_ dataset and the framework of the LOOCV. While the LOOCV was implemented for HGLDA, each known lncRNA-disease association was removed individually as a testing sample, and we further evaluated how well this test lncRNA-disease association ranked relative to the candidate sample. Here, the candidate samples comprised all potential lncRNA-disease associations without any known association evidence.

Thus, we could further obtain the corresponding true positive rates (TPR, sensitivity) and false positive rates (FPR, 1-specificity) by setting different thresholds. Here, sensitivity refers to the percentage of test samples that were predicted with ranks higher than the given threshold, and the specificity was computed as the percentage of negative samples with ranks lower than the threshold. The receiver-operating characteristic (ROC) curves were generated by plotting the TPR versus the FPR at different thresholds. Then, the AUCs were further calculated to evaluate the prediction performance of DCSMDA.

**Fig. 1:**
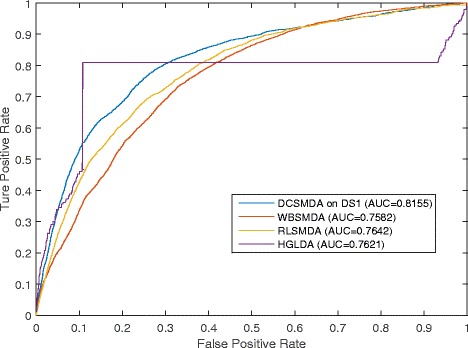
Performance comparisons between DCSMDA, RLSMDA and HGLDA in terms of ROC curve and AUC based on LOOCV

An AUC value of 1 represented a perfect prediction, while an AUC value of 0.5 indicated a purely random performance. The performance comparison in terms of the LOOCV results is shown in Fig. 1. In the LOOCV, the DCSMDA (when *b* was set to 6), RLSMDA, WBSMDA and HGLDA achieved AUCs of 0.8155, 0.7826, 0.7582 and 0.7621, respectively. DCSMDA predicted potential miRNA-disease associations without requiring known miRNA-disease associations. To the best of our knowledge, no methods that rely on known miRNA-disease associations exist. More importantly, considering that known disease-lncRNA associations remain very limited, the performance of DCSMDA can be further improved as additional known miRNA-disease associations are obtained in the future.

#### The stability analysis of DCSMDA

Because the current lncRNA-disease databases remain in their infancy and most existing methods are always evaluated using a specific dataset, the stability of the different datasets is ignored. To enhance the credibility of the prediction results, DCSMDA was further implemented using three different known lncRNA-disease association datasets, including *DS*_1_, *DS*_2_, and *DS*_3,_ and the known lncRNA-miRNA association dataset *DS*_4_.

**Fig. 2:**
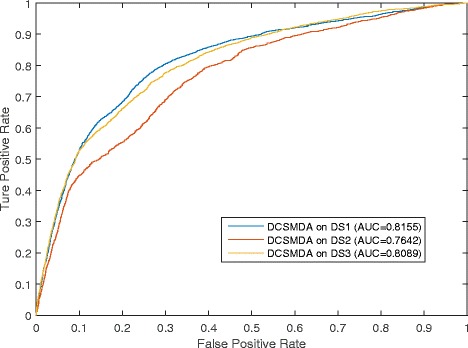
Comparison of different lncRNA-disease datasets to the prediction performance of DCSMDA

The comparison results of the ROC are shown in Fig. 2, and the corresponding AUCs are 0.8155, 0.8089 and 0.7642 when DCSMDA (*b* was set to 6) was evaluated in the framework of the LOOCV using the three different lncRNA-disease association datasets. DCSMDA achieved a reliable and effective prediction performance.

### Effects of the pre-given threshold parameter *b*

In DCSMDA, the pre-determined threshold *b* plays a critical role, and the value of *b* influences the performance of predicting potential miRNA-disease associations. In this section, we implemented a series of comparison experiments to evaluate the effects of *b* on the prediction performance of DCSMDA. The LOOCV was implemented, experiments were performed, and *b* was assigned different values. Considering the time complexity, and that the value of SPM(i, j) always equals 6, when *b* ≥6, we set *b* to a value no greater than 6 in our experiments.

**Fig. 3:**
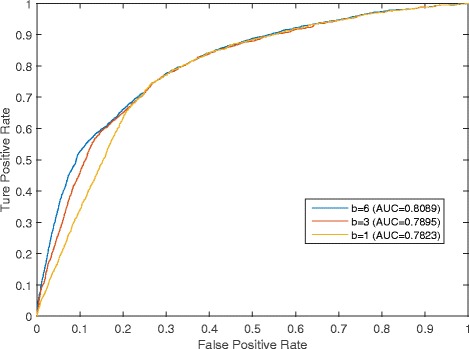
Comparison of effects of the pre-given threshold parameter *b* to the prediction performance of DCSMDA while *b* was assigned different values

As shown in Fig. 3, DCSMDA showed an increasing trend in its prediction performance as the value of the pre-determined threshold parameter *b* increased and achieved the best prediction performance when *b* was set to 6. When *b* was set to 6, DCSMDA achieved an AUC of 0.8089 using *DS*_3_ and *DS*_4_. In the analysis, we found that the main reason was that the number of known miRNA-lncRNA associations and lncRNA-disease associations was small; thus, when *b* is set to a larger value, more nodes could be linked to each other in the miRNA-lncRNA-disease interactive network, improving the prediction performance of DCSMDA. Therefore, we finally set *b* = 6 in our experiments.

## Case study

Currently, cancer is the leading cause of death in humans worldwide [34–36], and the incidence of cancer is high in both developed and developing countries. Therefore, to estimate the effective predictive performance of DCSMDA, case studies of two important cancers and leukaemia were implemented. The prediction results were verified using recently published experimental studies (see Table 1).

**Table 1:** DCSMDA was applied to case studies of three important cancers. In total, 10, 9 and 8 of the top 10 predicted pairs for these diseases were confirmed based on recent experimental studies

Disease	miRNA	Evidence (PMID and PMCID)
Prostatic Neoplasms	hsa-mir-15a	PMID: 25418933
Prostatic Neoplasms	hsa-mir-15b	PMID: 24661838
Prostatic Neoplasms	hsa-mir-16	PMID: 21880514
Prostatic Neoplasms	hsa-mir-195	PMID: 26080838
Prostatic Neoplasms	hsa-mir-424	PMID: 27820701
Prostatic Neoplasms	hsa-mir-497	PMID: 23886135
Prostatic Neoplasms	hsa-mir-125a	PMCID: PMC3979818
Prostatic Neoplasms	hsa-mir-106b	PMID: 26124181
Prostatic Neoplasms	hsa-mir-17	PMCID: PMC3008681
Prostatic Neoplasms	hsa-mir-93	PMID: 26124181
Lung Neoplasms	hsa-mir-15a	PMID: 26314859
Lung Neoplasms	hsa-mir-195	PMID: 25840419
Lung Neoplasms	hsa-mir-424	PMID: 27666545
Lung Neoplasms	hsa-mir-497	PMCID: PMC4537005
Lung Neoplasms	hsa-mir-16	PMID: 21192009
Lung Neoplasms	hsa-mir-15b	Unconfirmed
Lung Neoplasms	hsa-mir-125a	PMID: 24044511
Lung Neoplasms	hsa-mir-106a	PMID: 18328430
Lung Neoplasms	hsa-mir-106b	PMID: 18328430
Lung Neoplasms	hsa-mir-93	PMID: 24037530
Leukaemia	hsa-mir-424	PMID: 27013583
Leukaemia	hsa-mir-195	PMCID: PMC4713510
Leukaemia	hsa-mir-16	PMID:22912766
Leukaemia	hsa-mir-15a	PMID: 24392455
Leukaemia	hsa-mir-15b	PMCID: PMC4577143
Leukaemia	hsa-mir-497	Unconfirmed
Leukaemia	hsa-mir-125a	PMID: 22456625
Leukaemia	hsa-mir-19b	PMID: 28765931
Leukaemia	hsa-mir-19a	PMID: 28765931
Leukaemia	hsa-mir-17	PMID: 20439436

Prostate cancer (prostatic neoplasms), which is the second leading cause of cancer-related death in males, is among the most common malignant cancers and the most commonly diagnosed cancer in men worldwide. In 2012, prostate cancer occurred in 1.1 million men and caused 307,000 deaths. Accumulating evidence shows that microRNAs are strongly associated with prostate cancer. Therefore, DCSMDA was implemented to predict potential prostate cancer-related miRNAs. Consequently, ten of the top ten predicted prostate cancer-related miRNAs were validated by recent biological experimental studies (see Table 1). For example, Junfeng Jiang et al. reconstructed five prostate cancer co-expressed modules using functional gene sets defined by Gene Ontology (GO) annotation (biological process, GO_BP) and found that hsa-mir15a (ranked 1st) regulated these five candidate modules [37]. Medina-Villaamil V et al. analyzed circulating miRNAs in whole blood as non-invasive markers in patients with localized prostate cancer and healthy individuals and found that hsa-mir-15b (ranked 2nd) showed a statistically significant differential expression between the different risk groups and healthy controls [38]. Furthermore, Chao Cai et al. confirmed the tumour suppressive role of hsa-mir-195 (ranked 4th) using prostate cancer cell invasion, migration and apoptosis assays in vitro and tumour xenograft growth, angiogenesis and invasion in vivo by performing both gain-of-function and loss-of-function experiments [39].

Lung cancer (lung neoplasms) has the poorest prognosis among cancers and is the largest threat to people’s health and life. The incidence and mortality of lung cancer are rapidly increasing in China, and approximately 1.4 million deaths are due to lung cancer annually. Recent studies show that miRNAs play critical roles in the progression of lung cancer. Therefore, we used lung cancer as a case study and implemented DCSMDA; nine predicted lung cancer-associated miRNAs of the top ten prediction list were verified based on experimental reports. For example, Bozok Çetintaş V et al. analyzed the effects of selected miRNAs on the development of cisplatin resistance and found that hsa-mir-15a (ranked 1st) was among the most significantly downregulated miRNAs conferring resistance to cisplatin in Calu1 epidermoid lung carcinoma cells [40]. Hsa-mir-195, which ranked 2nd, was further confirmed to suppress tumour growth and was associated with better survival outcomes in several malignancies, including lung cancer [41]. Additionally, according to the biological experiments reported in several studies, hsa-mir-424 (ranked 3rd) plays an important role in lung cancer [42].

Leukaemia refers to a group of diseases that usually begin in the bone marrow and result in high numbers of abnormal white blood cells. The exact cause of leukaemia is unknown, and a combination of genetic factors and environmental factors is believed to play a role. In 2015, leukaemia presented in 2.3 million people and caused 353,500 deaths. Several studies suggest that miRNAs are effective prognostic biomarkers in leukaemia. For example, independent experimental observations showed relatively lower expression levels of mir-424 (ranked 1st) in TRAIL-resistant and semi-resistant acute myeloid leukaemia (AML) cell lines and newly diagnosed patient samples. The overexpression of mir-424 by targeting the 3′ UTR of PLAG1 enhanced TRAIL sensitivity in AML cells [43]. Hsa-mir-16 ranked 3rd, its expression was inversely correlated with Bcl2 expression in leukaemia, and both microRNAs negatively regulate B cell lymphoma 2 (Bcl2) at a posttranscriptional level. Bcl2 repression by these microRNAs induces apoptosis in a leukaemic cell line model [44]. The lncRNA H19 is considered an independent prognostic marker in patients with tumours. The expression of lncRNA H19 is significantly upregulated in bone marrow samples from patients with AML-M2. The results of the current study suggest that lncRNA H19 regulates the expression of inhibitor of DNA binding 2 (ID2) by competitively binding to hsa-mir-19b (ranked 8) and hsa-mir-19a (ranked 9), which may play a role in AML cell proliferation [45].

In addition, DCSMDA predicted all potential associations between the diseases and miRNAs in G_3_ simultaneously. In addition, notably, potential associations with a high predicted value can be publicly released and benefit from biological experimental validation. To further illustrate the effective performance of DCSMDA, the predicted results were sorted from best to worse, and the top 10 results were selected for analysis (see Table 2). Consequently, 100% of the results were confirmed by recent biological experiments and the HMDD dataset, and thus, DCSMDA can be used as an efficient computational tool in biomedical research studies.

**Table 2:** The top 10 predicted miRNA-disease associations by DCSMDA

Disease	MiRNA	Evidence
Carcinoma, Hepatocellular	hsa-mir-15a	HMDD
Carcinoma, Hepatocellular	hsa-mir-15b	HMDD
Carcinoma, Hepatocellular	hsa-mir-16	HMDD
Carcinoma, Hepatocellular	hsa-mir-195	HMDD
Carcinoma, Hepatocellular	hsa-mir-424	PMID: 26823812
Carcinoma, Hepatocellular	hsa-mir-497	HMDD
Colorectal Neoplasms	hsa-mir-497	HMDD
Colorectal Neoplasms	hsa-mir-15b	PMID: 23267864
Colorectal Neoplasms	hsa-mir-16	HMDD
Colorectal Neoplasms	hsa-mir-195	HMDD

## Discussion

Accumulating evidence shows that miRNAs play a very important role in several key biological functions and signalling pathways. A large-scale systematic analysis of miRNA-disease data performed by combining relevant biological data is highly important for humans and attractive topics in the field of computational biology. However, only a few prediction models have been proposed for the large-scale forecasting of associations between miRNAs and diseases based on lncRNA information. To utilize the wealth of disease-lncRNA, miRNA-lncRNA and disease-lncRNA association data recorded in four datasets and recently published experimental studies, in this article, we proposed a novel prediction model called DCSMDA to infer the potential associations between diseases and miRNAs. We first constructed a miRNA-lncRNA-disease interactive network and further integrated a distance correlation set, disease semantic similarity, functional similarity and Gaussian interaction profile kernel similarity for DCSMDA. The important difference between DCSMDA and previous computational models is that DCSMDA does not rely on any known miRNA-disease associations and predicts disease-miRNA associations based only on known disease-lncRNA associations and known lncRNA-miRNA associations. To evaluate the prediction performance of DCSMDA, the validation frameworks of the LOOCV were implemented using the HMDD database. Furthermore, case studies were further implemented using three important diseases and the top 10 predicted miRNA-disease associations based on recently published experimental studies and databases. The simulation results showed that DCSMDA achieved a reliable and effective prediction performance. Hence, DCSMDA could be used as an effective and important biological tool that benefits the early diagnosis and treatment of diseases and improves human health in the future.

However, although DCSMDA is a powerful method for predicting novel relationships between diseases and miRNAs, there are several limitations in our method. First, the value of the threshold parameter *b* plays an important role in DCSMDA, and the selection of a suitable value for *b* is a critical problem that should be addressed in future studies. Second, although DCSMDA does not rely on any known experimentally verified miRNA-disease relationships, the performance of DCSMDA was not very satisfactory compared with that of several existing methods, such as LRSMDA and WBSMDA [27, 28]. Introducing more reliable measures for the calculations of the disease similarity, miRNA similarity, and lncRNA similarity and developing a more reliable similarity integration method could improve the performance of DCSMDA. Finally, DCSMDA cannot be applied to unknown diseases or miRNAs that are not present in the disease-miRNA or lncRNA-miRNA databases; such genes are poorly investigated and have no known disease-lncRNA and lncRNA-miRNA associations. The performance of DCSMDA will be further improved once more known associations are obtained.

## Conclusion

In this article, we mainly achieved the following contributions: (1) we constructed a miRNA-lncRNA-disease interactive network based on common assumptions that similar diseases tend to show similar interaction and non-interaction patterns with lncRNAs, and similar miRNAs tend to show similar interaction and non-interaction patterns with lncRNAs; (2) the concept of a distance correlation set was introduced; (3) the sematic disease similarity, functionally similarity (including disease functionally similarity and miRNA functionally similarity) and Gaussian interaction profile kernel similarity (including disease Gaussian interaction profile kernel similarity, miRNA Gaussian interaction profile kernel similarity and lncRNA Gaussian interaction profile kernel similarity) were integrated; (4) the concept of an optimized matrix was introduced by integrating the Gaussian interaction profile kernel similarity of the miRNA pairs and disease pairs; (5) negative samples are not required in DCSMDA; and (6) DCSMDA can be applied to human diseases without relying on any known miRNA-disease associations.

## Methods

### Known disease-lncRNA associations

Because the number of lncRNA-disease associations is limited and many heterogeneous biological datasets have been constructed, we collected 8842 known disease-lncRNA associations from the MNDR dataset (http://www.bioinformatics.ac.cn/mndr/index.html) and 2934 known disease-lncRNA associations from the LncRNADisease dataset (http://www.cuilab.cn/lncrnadisease). Since the disease names in the LncRNADisease database differ from those in the MNDR dataset, we mapped the diseases in these two disease-lncRNA association datasets to their MeSH descriptors. After eliminating diseases without any MeSH descriptors, merging the diseases with the same MeSH descriptors and removing the lncRNAs that were not present in the lncRNA-miRNA dataset (*DS*_4_) used in this paper, 583 known lncRNA-disease associations (*DS*_1_) were obtained from the LncRNADisease dataset (see Additional file 1), and 702 known lncRNA-disease associations (*DS*_2_) were obtained from the MNDR dataset (see Additional file 2). Furthermore, after integrating the *DS*_1_ and *DS*_2_ datasets and removing the duplicate associations, we obtained the *DS*_3_ dataset, which included 1073 disease-lncRNA associations (see Additional file 3).

### Known lncRNA-miRNA associations

To construct the lncRNA-miRNA network, the lncRNA-miRNA association dataset *DS*_4_ was obtained from the starBasev2.0 database (http://starbase.sysu.edu.cn/) in February 2, 2017 and provided the most comprehensive experimentally confirmed lncRNA-miRNA interactions based on large-scale CLIP-Seq data. After the data pre-processing (including the elimination of duplicate values, erroneous data, and disorganized data), removing the lncRNAs that did not exist in the *DS*_3_ dataset and merging the miRNA copies that produced the same mature miRNA, we finally obtained 1883 lncRNA-miRNA associations (*DS*_4_) (see Additional file 4).

### Known disease-miRNA associations

To validate the performance of DCSMDA, the known human miRNA-disease associations were downloaded from the latest version of the HMDD database, which is considered the golden-standard dataset. In this dataset, after eliminating the duplicate associations and miRNA-disease associations involved with other diseases or lncRNAs not contained in the *DS*_3_ or *DS*_*4*_, we finally obtained 3252 high-quality lncRNA-disease associations (*DS*_5_) (see Additional file 5).

### Construction of the disease-lncRNA-miRNA interaction network

To clearly demonstrate the process of constructing the disease-lncRNA-miRNA interaction network, we use the disease-lncRNA dataset *DS*_3_ and the lncRNA-miRNA dataset *DS*_4_ as examples. We defined *L* to represent all the different lncRNA terms in *DS*_3_ and *DS*_4_ and then constructed the disease-lncRNA-miRNA interactive network based on *DS*_3_ and *DS*_4_ according to the following 3 steps:

Step 1 (Construction of the disease-lncRNA network): Let *D* and *L* be the number of different diseases and lncRNAs obtained from *DS*_3_, respectively. *S*_*D*_ = {*d*_*1*_*, d*_*2*_*,..., d*_*D*_} represents the set of all *D* different diseases in *DS*_3_. *S*_*L*_ = {*l*_*1*_*, l*_*2*_*,..., l*_*L*_} represents the set of all *L* different lncRNAs in *DS*_3_, and for any given *d*_*i*_ ∈ *S*_*D*_ and *l*_*j*_∈*S*_*L*_, we can construct the D*L dimensional matrix KAM1 as follows:

1 $$ KAM1\left(i,j\right)=\Big\{{\displaystyle \begin{array}{c}1\kern0.5em if\kern0.2em {d}_i\kern0.2em is\kern0.34em related\kern0.34em to\kern0.2em {l}_j\kern0.2em in\kern0.2em {DS}_3\\ {}0\kern7.8em otherwise\end{array}} $$

Step 2 (Construction of the lncRNA-miRNA network): Let *M* be the number of different miRNAs obtained from *DS*_4_. *S*_*M*_ = {*m*_*1*_*, m*_*2*_*,..., m*_*M*_} represents the set of all *M* different miRNAs in *DS*_4_, and for any given *m*_*i*_∈*S*_*M*_ and *l*_*j*_∈*S*_*L*_, we can construct the M*L dimensional matrix *KAM2* as follows:

2 $$ KAM2\left(i,j\right)=\left\{\begin{array}{c}1\kern0.5em if\ {m}_i\  is\ related\ to\ {l}_j\  in\ {DS}_4\\ {}0\kern5.25em otherwise\end{array}\right. $$

Step 3 (Constriction of the disease-lncRNA-miRNA interactive network): Based on the disease-lncRNA network and lncRNA-miRNA network, we can obtain the undirected graph *G*_*3*_ *=* (*V*_*3*_*, E*_*3*_), where *V*_*3*_ = *S*_*D*_ ∪*S*_*L*_ ∪*S*_*M*_ = {*d*_*1*_*, d*_*2,*_*..., d*_*D*_*, l*_*D + 1*_*, l*_*D + 2*_*..., l*_*D + L*_*, m*_*D + L + 1*_*, m*_*D + L + 2*_*..., m*_*D + L + M*_} is the set of vertices, *E*_*3*_ is the edge set of *G*_*3*_, and *d*_*i*_∈*S*_*D*_, *l*_*j*_∈*S*_*L*_, m_k_∈S_M_. Here, an edge exists between *d*_*i*_ and *l*_*j*_ in *E*_*3*_*KAM1*(*d*_*i*_, *l*_*j*_) = 1, an edge exists between *l*_*j*_ and *m*_*k*_ in *E*_*3*_ if *KAM2*(*m*_*k*_*, l*_*j*_) = 1. Then, for any given *a*, *b*∈*V*_*3*_, we can define the Strong Correlation (*SC*) between *a* and *b* as follows:

3 $$ SC\left(a,b\right)=\left\{\begin{array}{c}1\kern0.5em if\kern0.34em there\kern0.34em is\kern0.34em an\kern0.34em edge\kern0.34em between\kern0.2em a\kern0.2em and\kern0.2em b\\ {}0\kern11em otherwise\end{array}\right. $$

Notably, although we did not use any known disease-miRNA associations, the diseases and miRNAs can still be indirectly linked by integrating the edges between the disease nodes, the lncRNA nodes and edges between the miRNA nodes and lncRNA nodes in *G*_*3*_.

### Disease semantic similarity

**Fig. 4:**
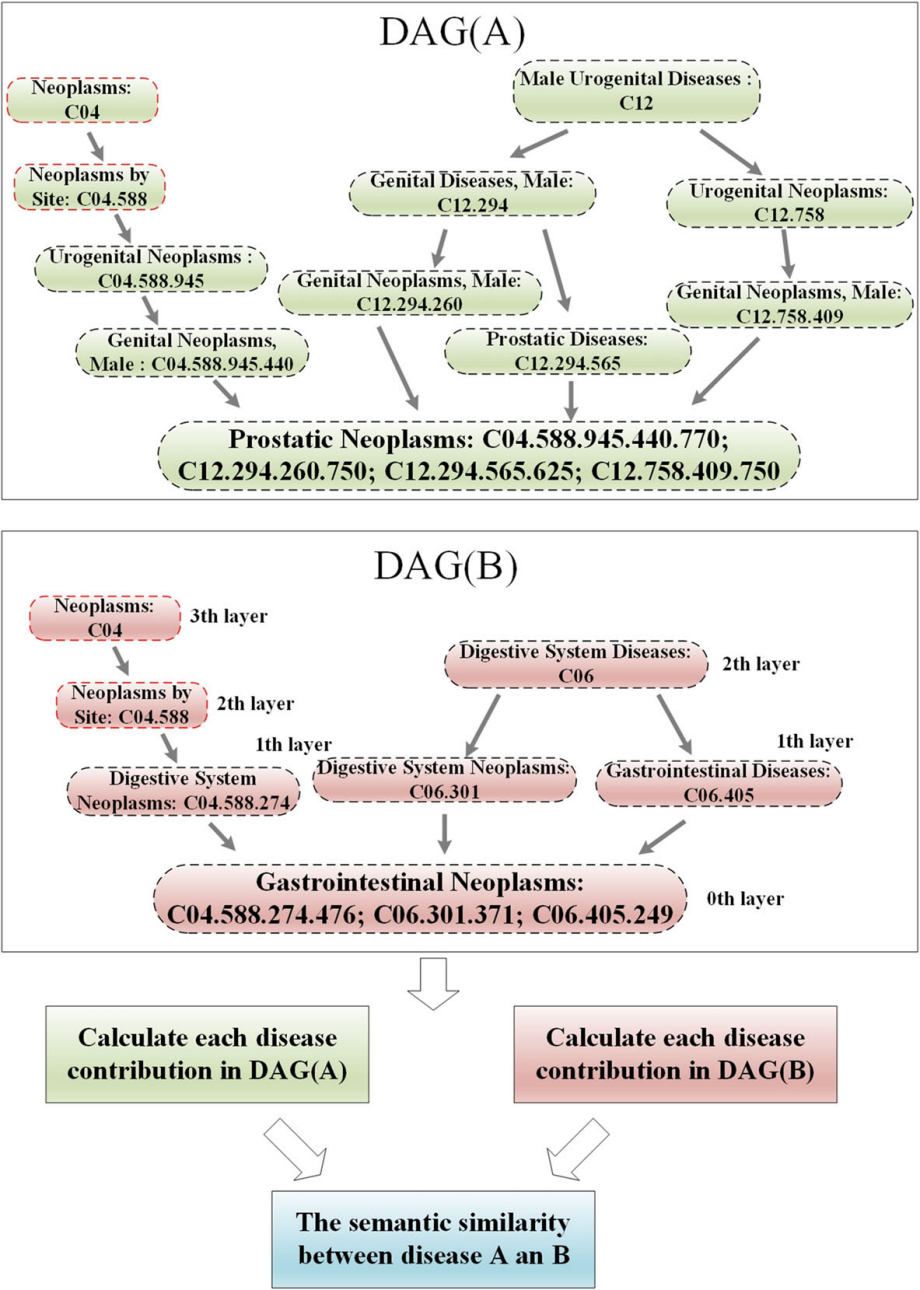
The disease *DAGs* of Prostatic Neoplasms and Gastrointestinal Neoplasms

We downloaded the MeSH descriptors of the diseases from the National Library of Medicine (http://www.nlm.nih.gov/), which introduced the concept of Categories and Subcategories and provided a strict system for disease classification. The topology of each disease was visualized as a Directed Acyclic Graph (DAG) in which the nodes represented the disease MeSH descriptors, and all MeSH descriptors in the DAG were linked from more general terms (parent nodes) to more specific terms (child nodes) by a direct edge (see Fig. 4). Let *DAG(A)* = (*A, T*(*A*)*, E*(*A*)), where *A* represents disease *A*, *T*(*A*) represents the node set, including node *A* and its ancestor nodes, and *E*(*A*) represents the corresponding edge set. Then, we defined the contribution of disease term *d* in *DAG*(*A*) to the semantic value of disease *A* as follows:

4 $$ \left\{\begin{array}{c}{D}_A(d)=1\kern16.8em if\kern0.3em d=A\\ {}{D}_A(d)=\max \left\{0.5\ast {D}_A\left({d}^{\ast}\right)|{d}^{\ast}\in children\kern0.3em of\kern0.3em d\right\}\kern0.3em if\kern0.3em d\ne A\end{array}\right. $$

For example, the semantic value of the disease ‘Gastrointestinal Neoplasms’ shown in Fig. 4 is calculated by summing the weighted contribution of ‘Neoplasms’ (0.125), ‘Neoplasms by Site’ (0.25), ‘Digestive System Diseases’ (0.25), ‘Digestive System Neoplasms’ (0.5), ‘Digestive System Neoplasms’ (0.5) and ‘Gastrointestinal Diseases’ (0.5) to ‘Gastrointestinal Neoplasms’ and the contribution to ‘Gastrointestinal Neoplasms’ (1) by ‘Gastrointestinal Neoplasms’.

Then, the sematic value of disease *A* can be obtained by summing the contribution from all disease terms in = *DAG*(*A*), and the semantic similarity between the two diseases *d*_*i*_ and *d*_*j*_ can be calculated as follows:

5 $$ SSD\left({d}_i,{d}_j\right)=\frac{\sum \limits_{d\in \left(T\left({d}_i\right)\cap T\left({d}_j\right)\right)}\left({D}_{d_i}(d)+{D}_{d_j}(d)\right)}{\sum \limits_{d\in T\left({d}_i\right)}{D}_{d_i}(d)+{\sum}_{d\in T\left({d}_j\right)}{D}_{d_j}(d)} $$

where *SSD* is the disease semantic similarity matrix.

### MiRNA Gaussian interaction profile kernel similarity

Based on the assumption that similar miRNAs tend to show similar interaction and non-interaction patterns with lncRNAs, in this section, we introduce the Gaussian interaction profile kernel used to calculate the network topologic similarity between miRNAs and used the vector *MLP*(*m*_*i*_) to denote the ith row of the adjacency matrix *KAM2*. Then, the Gaussian interaction profile kernel similarity for all investigated miRNAs can be calculated as follows:

6 $$ MGS\left({m}_i,{m}_j\right)=\exp \left(-\frac{M\ast {\left\Vert MLP\left({m}_i\right)- MLP\left({m}_j\right)\right\Vert}^2}{\sum \limits_{i=1}^M{\left\Vert MLP\left({m}_i\right)\right\Vert}^2}\right) $$

where parameter *M* is the number of miRNAs in *DS*_4_.

### Disease Gaussian interaction profile kernel similarity

Based on the assumption that similar diseases tend to show similar interaction and non-interaction patterns with lncRNAs, the Gaussian interaction profile kernel similarity for all investigated diseases can be calculated as follows:

7 $$ DGS\left({d}_i,{d}_j\right)=\exp \left(-\frac{D\ast {\left\Vert DLP\left({d}_i\right)- DLP\left({d}_j\right)\right\Vert}^2}{\sum \limits_{i=1}^D{\left\Vert DLP\left({d}_i\right)\right\Vert}^2}\right) $$

where parameter *D* is the number of diseases in *DS*_3,_ and *DLP*(*d*_*i*_) represent the ith row of the matrix *KAM1*. Then, based on previous work [46], we can improve the predictive accuracy problems by logistic function transformation as follows:

8 $$ FDGS\left({d}_i,{d}_j\right)=\frac{1}{1+{e}^{-15\ast DGS\left({d}_i,{d}_j\right)+\log (9999)}} $$

### lncRNA Gaussian interaction profile kernel similarity

Based on the assumption that similar lncRNAs tend to show similar interaction and non-interaction patterns with miRNAs and similar lncRNAs tend to show similar interaction and non-interaction patterns with diseases, the Gaussian interaction profile kernel similarity matrix for all investigated lncRNAs in *DS*_3_ can be computed in a similar way as that for disease, as follows:

9 $$ LGS1\left({l}_i,{l}_j\right)=\exp \left(-\frac{L\ast {\left\Vert LDP\left({l}_i\right)- LDP\left({l}_j\right)\right\Vert}^2}{\sum \limits_{i=1}^L{\left\Vert LDP\left({l}_i\right)\right\Vert}^2}\right) $$

where parameter *L* is the number of lncRNAs in *DS*_3,_ and *LDP*(*l*_*i*_) represents the ith column of the matrix *KAM1*.

Obviously, the Gaussian interaction profile kernel similarity for all investigated lncRNAs in *DS*_4_ can be computed as follows:

10 $$ LGS2\left({d}_i,{d}_j\right)=\exp \left(-\frac{L\ast \parallel LMP\left({l}_i\right)- LMP\left({l}_j\right){\parallel}^2}{\sum \limits_{i=1}^L\parallel LMP\left({l}_i\right){\parallel}^2}\right) $$

where *LMP*(*l*_*i*_) represents the ith column of the matrix *KAM2*.

### Disease functional similarity based on the lncRNAs

**Fig. 5:**
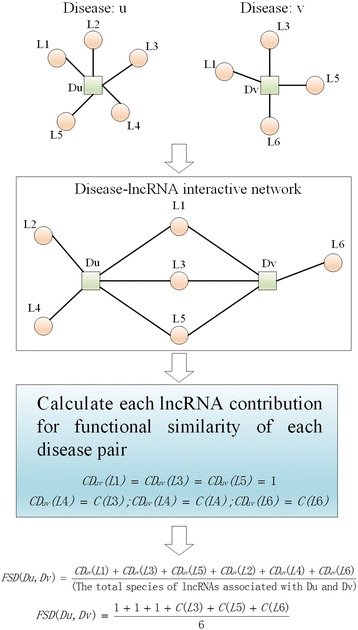
The Flow chart of the disease functional similarity calculation model

To calculate the functional similarity of the diseases, we first constructed the undirected graph *G*_*1*_ = (*V*_*1*_*, E*_*1*_) based on *KAM1*, where *V*_*1*_ = *S*_*D*_∪*S*_*M*_ = {*d*_*1*_*, d*_*2*_*, …, d*_*D*_*, l*_*D + 1*_*, l*_*D + 2*_*,…, l*_*D + M*_} is the set of vertices, *E*_*1*_ is the set of edges, and for any two nodes *a, b*∈*V*_*1*_, an edge exists between a and b in *E*_*1*_ if *KAM1*(*a, b*) = 1. Therefore, we can calculate the similarities between two disease nodes by comparing and integrating the similarities of the lncRNA nodes associated with these two disease nodes based on the assumption that similar diseases tend to show similar interaction and non-interaction patterns with lncRNAs. The procedure used to calculate the disease functional similarity is shown in Fig. 5.

Because different lncRNA terms in *DS*_3_ may relate to several diseases, assigning the same contribution value to all miRNAs is not suitable, and therefore, we defined the contribution value of each lncRNA as follows:

11 $$ C\left({l}_i\right)=\frac{\mathrm{The}\kern0.34em \mathrm{number}\kern0.34em \mathrm{of}\kern0.2em {l}_i-\mathrm{related}\kern0.34em \mathrm{edges}\ \mathrm{in}\ {E}_1}{\mathrm{The}\ \mathrm{number}\ \mathrm{of}\ \mathrm{all}\ \mathrm{edges}\ \mathrm{in}\ {E}_1} $$

Based on the definition of *C*(*l*_*i*_), we can define the contribution value of each lncRNA to the functional similarity of each disease pair as follows:

12 $$ {CD}_{ij}\left({l}_k\right)=\Big\{{\displaystyle \begin{array}{c}1\kern2.30em if\kern0.3em lncRNA\kern0.3em {l}_k\kern0.2em related\kern0.34em to\kern0.2em {d}_i\kern0.2em and\kern0.2em {d}_j\kern0.2em simultaneously\\ {}C\left({l}_k\right)\kern6em if\kern0.34em lncRNA\kern0.3em {l}_k\kern0.2em only\kern0.34em related\kern0.34em to\kern0.2em {d}_i\kern0.2em or\kern0.2em {d}_j\end{array}}\operatorname{} $$

Finally, we can define the functional similarity between diseases *d*_*i*_ and *d*_j_ by integrating lncRNAs related to *d*_*i*_*, d*_*j*_ or both as follows:

13 $$ FSD\left({d}_i,{d}_j\right)=\frac{\sum \limits_{l_k\in \left(D\left({d}_i\right)\cup D\left({d}_j\right)\right)}C{D}_{ij}\left({l}_k\right)}{\mid D\left({d}_i\right)\mid +\mid D\left({d}_j\right)\mid -\mid D\left({d}_i\right)\cap D\left({d}_j\right)\mid } $$

where *D*(*d*_*i*_) and *D*(*d*_*j*_) represent all lncRNAs related to *di* and *d*_*j*_ in *E*_*1*_, respectively.

### MiRNA functional similarity based on lncRNAs

Based on the assumption that similar miRNAs tend to show similar interaction and non-interaction patterns with lncRNAs, we can also calculate the miRNA functional similarity in the lncRNA-miRNA interactive network. Similar to the procedure used to calculate the disease functional similarity, first, we constructed the undirected graph *G*_*2*_ = (*V*_*2*_*, E*_*2*_), where *V*_*2*_ = *S*_*M*_∪*S*_*L*_ = {*m*_*1*_*, m*_*2*_*,…, l*_*M + 1*_*, l*_*M + 2*_*,…, l*_*M + L*_} is the set of vertices, *E*_*2*_ is the set of edges, and for any two nodes *a, b* ∈ *V*_*2*_, an edge exists between *a* and *b* in *E*_*2*_ if *KAM2*(*a*, *b*) = 1. Then, we defined the contribution of each lncRNA to the functional similarity of each miRNA pair as follows:

14 $$ {CM}_{ij}\left({l}_k\right)=\Big\{{\displaystyle \begin{array}{c}1\kern1.20em if\kern0.34em lncRNA\kern0.3em {l}_k\kern0.2em related\kern0.2em {m}_i\kern0.2em and\kern0.2em {m}_j\kern0.2em simultaneously\\ {}C\left({l}_k\right)\kern5em if\kern0.34em lncRNA\kern0.3em {l}_k\kern0.2em only\kern0.34em related\kern0.2em {m}_i\kern0.2em or\kern0.2em {m}_j\end{array}}\operatorname{} $$

Additionally, we can define the functional similarity between *m*_*i*_ and *m*_*j*_ as follows:

15 $$ FSM\left({m}_i,{m}_j\right)=\frac{\sum \limits_{l_k\in \left(D\left({m}_i\right)\cup D\left({m}_j\right)\right)}C{M}_{ij}\left({m}_k\right)}{\mid D\left({m}_i\right)\mid +\mid \mathrm{D}\left({m}_j\right)\mid -\mid D\left({m}_i\right)\cap D\left({m}_j\right)\mid } $$

where *D*(*m*_*i*_) represents all lncRNAs related to *m*_*i*_, and *D*(*m*_*j*_) represents lncRNAs relate to *m*_*j*_ in *E*_*2*_.

### Integrated similarity

**Fig. 6:**
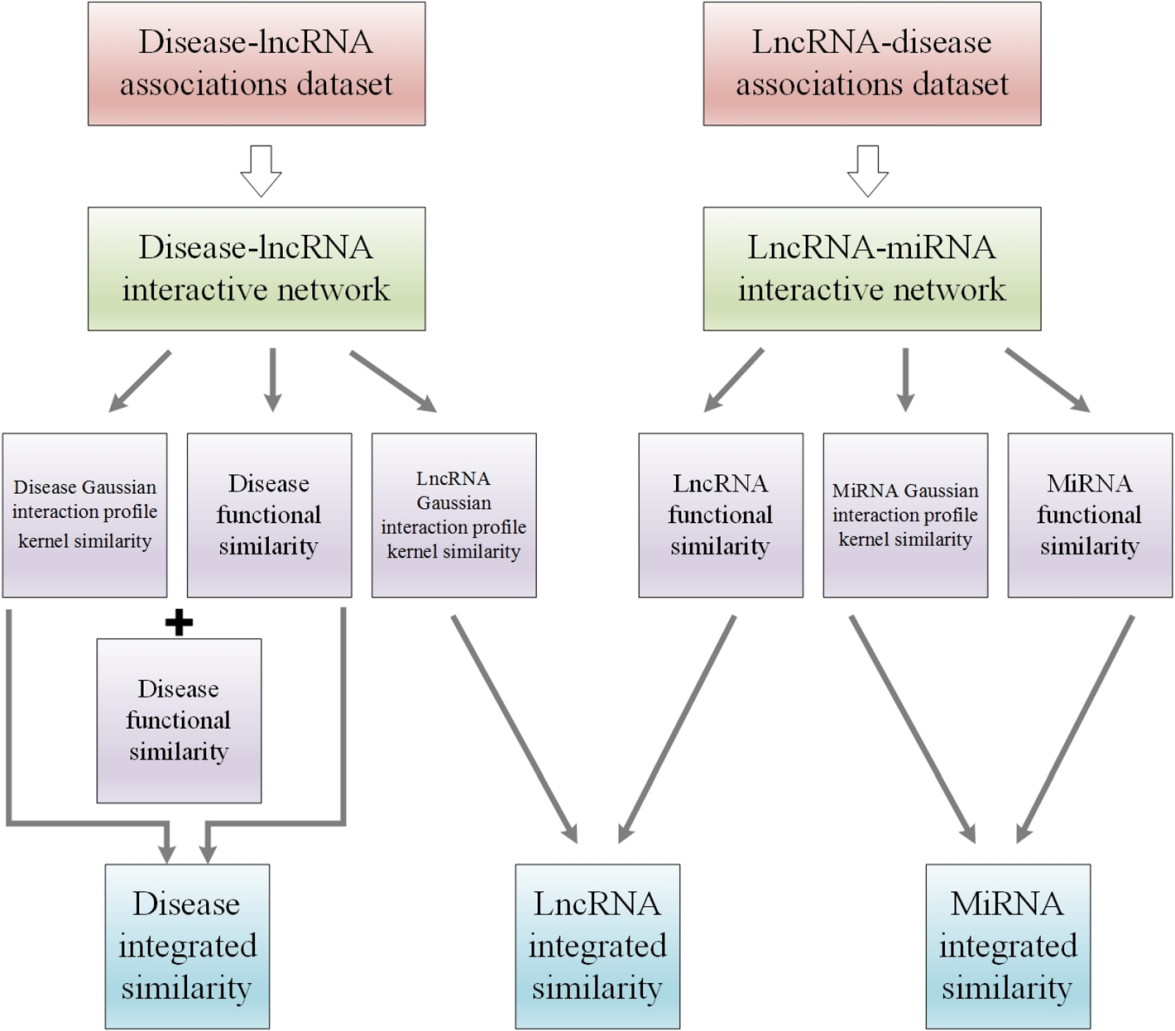
Flow chart of calculation of diseases integrated similarity, lncRNA integrated similarity and miRNA integrated similarity

The processes used to calculate the integrated similarities of the diseases, lncRNAs and miRNAs are illustrated in Fig. 6. Combining the disease semantic similarity, the disease Gaussian interaction profile kernel similarity and the disease functional similarity mentioned above, we can construct the disease integrated similarity matrix *FDD* as follows:

16 $$ FDD=\frac{SSD+ FDGS+ FSD}{3} $$

Additionally, based on the miRNA Gaussian interaction profile kernel similarity and the miRNA functional similarity, we can construct the miRNA integrated similarity matrix *FMM* as follows:

17 $$ FMM=\frac{MGS+ FSM}{2} $$

Furthermore, based on the Gaussian interaction profile kernel similarity matrices *LGS1* and *LGS2*, we can construct the lncRNA integrated similarity matrix *FLL* as follows:

18 $$ FLL=\frac{LGS1+ LGS2}{2} $$

### Prediction of disease-miRNA associations based on a distance correlation set

**Fig. 7:**
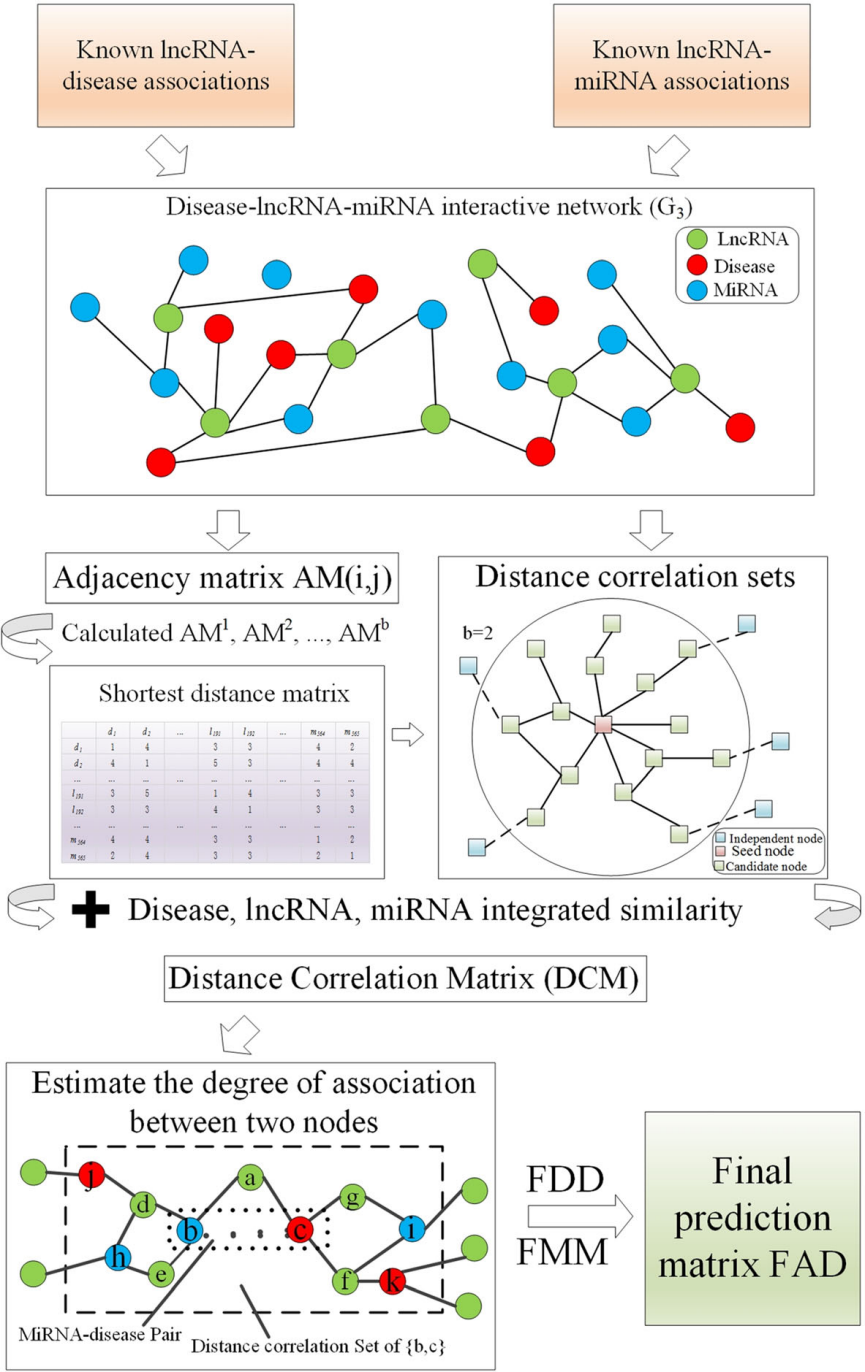
The procedures of DCSMDA

In this section, we developed a novel computational method, i.e., DCSMDA, to predict potential disease-miRNA associations by introducing a distance correlation set based on the following assumptions: similar diseases tend to show similar interaction and non-interaction patterns with lncRNAs, and similar lncRNAs tend to show similar interaction and non-interaction patterns with miRNAs. As illustrated in Fig. 7, the DCSMDA procedure consists of the following 5 major steps:

Step 1 (Construction of the adjacency matrix based on *G*_*3*_): First, we construct a (*D + L + M*) * (*D + L + M*) Adjacency Matrix (*AM*) based on the undirected graph *G*_*3*_ and *SC*, and then for any two nodes *v*_*i*_*, v*_*j*_∈*V*_*3*_*,* we can define the *AM*(*i, j*) as follows:

19 $$ AM\left(i,j\right)=\left\{\begin{array}{c} SC\left({d}_i,{d}_j\right),\kern0.75em if\kern0.5em i\in \left[1,D\right]\ \mathrm{and}\ j\in \left[1,D\right].\kern6.25em \\ {} SC\left({d}_i,{l}_j\right),\kern0.75em if\kern0.5em i\in \left[1,D\right]\ \mathrm{and}\kern0.5em j\in \left[D,D+L\right].\kern4.75em \\ {} SC\left({d}_i,{m}_j\right),\kern1.25em if\kern0.5em i\in \left[1,D\right]\ \mathrm{and}\ j\in \left[D+L,D+L+M\right].\kern3em \\ {} SC\left({m}_i,{d}_j\right),\kern1em if\kern0.5em i\in \left[D,D+L\right]\ \mathrm{and}\ j\in \left[1,D\right].\kern4.75em \\ {} SC\left({m}_i,{m}_j\right),\kern1.25em if\kern0.5em i\in \left[D,D+L\right]\ \mathrm{and}\ j\in \left[\mathrm{D},D+L\right].\kern3.25em \\ {} SC\left({m}_i,{l}_j\right),\kern1.25em if\kern0.5em i\in \left[D,D+L\right]\ \mathrm{and}\ j\in \left[D+L,D+L+M\right].\kern1.75em \\ {} SC\left({l}_i,{d}_j\right),\kern1.25em if\kern0.5em i\in \left[D+L,D+L+M\right]\ \mathrm{and}\ j\in \left[1,D\right].\kern3em \\ {} SC\left({l}_i,{m}_j\right),\kern1.25em if\kern0.5em i\in \left[D+L,D+L+M\right]\ \mathrm{and}\ j\in \left[\mathrm{D},D+L\right].\kern1.75em \\ {} SC\left({l}_i,{m}_j\right),\kern1.25em if\kern0.5em i\in \left[D+L,D+L+M\right]\ \mathrm{and}\ j\in \left[D+L,D+L+M\right]\end{array}\right. $$

where *i*∈[1*, D* + *L + M*] and *j*∈[1*, D + L* + *M*], and to calculate the shortest distance matrix in step 2, we define *AM* (*i, j*) = 1 if *i = j*.

Step 2 (Construction of the shortest distance matrix based on adjacency matrix *AM*): First, we set parameter *b* to control the bandwidth of the distance correlation set and let *b* be a pre-determined positive integer, and then, we can obtain *b* matrices, such as *AM*^*1*^*, AM*^*2*^*,..., AM*^*b*^, based on the above formula (19), and the Shortest Path Matrix is calculated as follows:

20 $$ SPM\left(i,j\right)=\left\{\ \begin{array}{c}1,\kern2.5em if\  AM\left(i,j\right)=1\\ {}k,\kern2.25em otherwise\kern1.25em \end{array}\right. $$

where *i*∈[1*, D* + M + *L*], *j*∈[1*, D* + M + *L*], *k*∈[2*, b*], and *k* satisfies the following: *AM* ^*k*^(*i*, *j*)≠0, while *AM* ^1^(*i*, *j*) = *AM* ^2^(*i*, *j*) = … = *AM* ^*k-*1^(*i*, *j*) = 0.

Step 3 (Calculation of distance correlation sets and distance coefficient of each node pair in *G*_*3*_):

For each node *v*_*i*_ ∈ *V*_*3*_, we can obtain distance correlation set *DCS*(*i*) according to the shortest distance matrix as follows:

21 $$ DCS(i)=\left\{{v}_j|r\ge SPM\left(i,j\right)>0\right\} $$

where *DCS*(*i*) of each node contains itself and all nodes with the shortest distance less than *b*.

For instance, in the disease-miRNA-lncRNA interaction network illustrated in Fig. 7, *DCS* (seed node) is all candidate nodes when *b* is set to 2.

Then, we can calculate the distance coefficient (*DC*) of the node pair (v_i_, v_j_) as follows:

22 $$ P\left(i,j\right)=\left\{\begin{array}{c} SPM{\left(i,j\right)}^{b+1}, if\ i\in DCS(j)\  or\ j\in DCS(i)\\ {}0,\kern3.5em otherwise\end{array}\right. $$

Furthermore, we can construct a Distance Correlation Matrix (*DCM*) based on the disease integrated similarity, the lncRNA integrated similarity, and the miRNA integrated similarity as follows:

23 $$ DCM\left(i,j\right)=\Big\{{\displaystyle \begin{array}{c}P\left(i,j\right)\ast \exp \left( FDD\left(i,j\right)\right),\kern7.9em if\kern0.5em i\in \left[1,D\right]\ \mathrm{and}\ j\in \left[1,D\right].\kern6.3em \\ {}P\left(i,j\right)\ast \exp \left( FLL\left(i,j\right)\right),\kern6em if\kern0.5em i\in \left[D,D+L\right]\ \mathrm{and}\ j\in \left[\mathrm{D},D+L\right].\kern4.75em \\ {}P\left(i,j\right)\ast \exp \left( FMM\left(i,j\right)\right),\kern0.5em if\kern0.5em i\in \left[D+L,D+L+M\right]\ \mathrm{and}\ j\in \left[D+L,D+L+M\right]\kern3em \\ {}P\left(\mathrm{i},\mathrm{j}\right)\ast \frac{SPM\left(i,j\right)}{b},\kern18.5em \mathrm{otherwise}\kern5.5em \end{array}}\operatorname{} $$

where *i*∈[1, *D + L + M*] and *j*∈[1, *D + L + M*].

Step 4 (Estimation of the association degree between a pair of nodes): Based on formula (23), we can estimate the association degree between v_i_ and v_j_ as follows:

24 $$ PM\left(i,j\right)=\frac{\sum \limits_{k=1}^{D+L+M} DCM\left(i,k\right)+{\sum}_{k=1}^{D+L+M} DCM\left(k,j\right)}{D+L+M} $$

Thus, we can obtain prediction matrix *PM*, where the entity *PM (i, j)* in row *i* column *j* represents the predicted association between node *v*_*i*_ and *v*_*j*_.

Step 5 (Calculation of the final prediction result matrix between the miRNAs and diseases): Let $$ PM=\left[\begin{array}{c}{C}_{11}\kern0.75em {C}_{12}\kern1em {C}_{13}\\ {}{C}_{21}\kern0.75em {C}_{22}\kern1em {C}_{23}\\ {}{C}_{31}\kern0.75em {C}_{32}\kern0.75em {C}_{33}\end{array}\right] $$, where *C*_11_ is a *D*×*D* matrix, *C*_12_ is a *D*×*L* matrix, *C*_13_ is a *D*×*M* matrix, *C*_21_ is an *L*×*D* matrix, *C*_*22*_ is an *L* ×*L* matrix, *C*_*23*_ is an L×M matrix, C_31_ is an M×D matrix, *C*_*32*_ is an *M*×*L* matrix and *C*_*33*_ is an *M* ×*M* matrix. Obviously, *C*_*13*_ is our predicted result, which provides the association probability between each disease and miRNA. A previous study [27] demonstrated that the Gaussian interaction profile kernel similarity is a high-efficiency tool for optimizing the result of prediction, and therefore, we used the miRNA Gaussian interaction profile kernel similarity and the disease Gaussian interaction profile kernel similarity to optimize the result of the DCSMDA as follows:

25 $$ FAD= FDD\ast {C}_{13}\ast FMM $$

where the matrix FAD denotes the relationship between the miRNA-disease pairs.

## Additional files

Additional file 1: The known lncRNA-disease associations for constructing the *DS*_1_. We list 583 known lncRNA-disease associations which were collected from LncRNAdisease dataset to construct the *DS*_1_. (XLS 58 kb)

Additional file 2: The known lncRNA-disease associations for constructing the *DS*_2_. We list 702 known lncRNA-disease associations which were collected from MNDR dataset to construct the *DS*_2_. (XLS 63 kb)

Additional file 3: The integrated lncRNA-disease associations for constructing the *DS*_3_. We list 1073 lncRNA-disease associations which were collected by integrating the datasets of *DS*_1_ and *DS*_2_. (XLS 83 kb)

Additional file 4: The known lncRNA-miRNA associations for constructing the *DS*_4_. We list 1883 known lncRNA-miRNA associations which were collected from starBasev2.0 database to construct the *DS*_4_. (XLS 123 kb)

Additional file 5: The known miRNA-disease associations for constructing the *DS*_5_. We list 3252 high-quality miRNA-disease associations which were collected from HMDD database to validate the performance of our method. (XLS 191 kb)

## Abbreviations

AUC: Areas under ROC curve
DCSMDA: Distance Correlation Set is developed to predict MiRNA-Disease Associations
FPR: False positive rates
GO: Gene Ontology
miRNA: MicroRNA
ncRNA: Noncoding RNA
ROC: Receiver-operating characteristics
TPR: True positive rates
LOOCV: Leave-One Out Cross Validation
